# A rare case of acute generalized exanthematous pustulosis-like Sweet syndrome

**DOI:** 10.1093/skinhd/vzag029

**Published:** 2026-04-20

**Authors:** Brandon C E Hu, Bowen Xia, Paul Curnow, Ian Simpson, Francis Y X Lai, Senhong Lee

**Affiliations:** Department of Dermatology, Monash Health, Clayton, VIC, Australia; Department of Dermatology, Monash Health, Clayton, VIC, Australia; Department of Dermatology, Monash Health, Clayton, VIC, Australia; Department of Dermatology, Royal Children’s Hospital Melbourne, Parkville, VIC, Australia; Department of Pathology, Monash Health, Clayton, VIC, Australia; Department of Dermatology, Monash Health, Clayton, VIC, Australia; Skin Health Institute, Carlton, VIC, Australia; Department of Dermatology, Monash Health, Clayton, VIC, Australia; Skin Health Institute, Carlton, VIC, Australia; Department of Dermatology, Eastern Health, Box Hill, VIC, Australia; School of Clinical Sciences, Faculty of Medicine, Nursing and Health Sciences, Monash University, Melbourne, VIC, Australia

## Abstract

Acute generalized exanthematous pustulosis (AGEP) is a pustular drug eruption characterized by superficial pustules, commonly caused by β-lactam antibiotics. Management of AGEP involves discontinuation of the offending medication and supportive therapy with topical corticosteroids and analgesia, given the self-limiting nature of this condition. In contrast, Sweet syndrome (SS), or acute febrile neutrophilic dermatosis, is characterized by the sudden onset of painful, inflamed skin lesions associated with fever. SS typically requires investigations for associated conditions and systemic corticosteroids, resulting in rapid improvement within days. We present a case of AGEP-like SS, initially diagnosed clinically as AGEP in the setting of intravenous antibiotic use, where the patient deteriorated, despite antibiotics being stopped. Skin biopsy revealed papillary dermal oedema with neutrophilic infiltrate, and scattered histiocytoid cells and eosinophils, resulting in a revised diagnosis of AGEP-like SS. The patient was started on oral corticosteroids and achieved complete resolution of disease within 2 days. This case highlights AGEP-like SS as an important differential diagnosis in patients presenting with AGEP-like eruptions, as the management of AGEP and SS is different.

## Teaser

Sweet syndrome is a neutrophilic dermatosis characterized by fever and the acute onset of tender, erythematous plaques or nodules. While the pustular variant of Sweet syndrome is well recognized, the acute generalized exanthematous pustulosis (AGEP)-like presentation is rarely reported. We report a case AGEP-like Sweet syndrome of the histiocytoid variant.

What is already known about this topic?

- Acute generalized exanthematous pustulosis (AGEP) is a pustular drug eruption that typically resolves following cessation of the offending agent.
- Sweet syndrome (SS) is a neutrophilic dermatosis with heterogeneous clinical and histological variants.
- Although AGEP and SS may share overlapping clinical features, they differ significantly in their underlying pathology and management.

What does this study add?

- While the pustular variant of SS is well recognized, AGEP-like SS is not a well-established entity.
- This report describes a case of AGEP-like SS, highlighting the importance of distinguishing it from AGEP to ensure timely initiation of systemic therapy and investigations for associated condition.

Sweet syndrome (SS) is a neutrophilic dermatosis characterized by fever and the acute onset of tender, erythematous plaques or nodules. While the pustular variant of SS is well recognized, the acute generalized exanthematous pustulosis (AGEP)-like presentation is rarely reported. We report a case of AGEP-like SS of the histiocytoid variant.

## Case report

**Figure 1:**
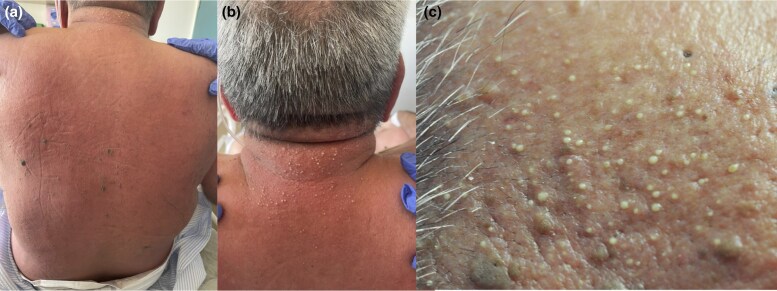
(a) Multiple nonfolliculocentric pustules on a background of confluent erythema and oedema affecting the back and neck. (b) Close-up view of nonfolliculocentric pustules on the posterior neck. (c) Close-up view of pustules on the right forehead, some of which are nonfolliculocentric.

A 72-year-old man admitted for ischaemic stroke was referred with a generalized pustular eruption associated with high-grade fever (40.3 °C). This occurred 1 day following treatment of presumed aspiration pneumonia with intravenous ceftriaxone, azithromycin and metronidazole. On examination, nonfolliculocentric pustules were noted, affecting his face, neck and trunk, with associated confluent erythema and oedema; there was sparing of his mucocutaneous surfaces (Figure 1). He was diagnosed clinically with AGEP, following the acute onset and exposure to antibiotics. However, the patient continued to deteriorate, despite cessation of all new medications, and was started on oral prednisolone 50 mg daily, owing to the critical nature of his disease. An infective and rheumatological screen was conducted, including computed tomography of the chest, abdomen and pelvis. The results were negative except for a neutrophilia [22.12 × 10^9^ cells L^–1^; reference interval (RI) 2.00–8.00 × 10^9^ cells L^–1^] and elevated C-reactive protein (299 mg L^–1^; RI 0–5 mg L^–1^). Multiple punch biopsies were taken for histopathological assessment.

**Figure 2:**
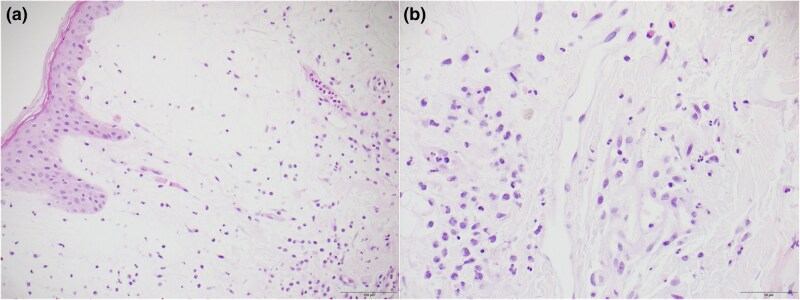
(a) Papillary dermal oedema with neutrophilic infiltrate, as well as scattered mononuclear histiocytoid cells and eosinophils (haematoxylin and eosin, ×20). (b) Neutrophilic infiltrate admixed with mononuclear histiocytoid cells and eosinophils (haematoxylin and eosin, ×40).

Histopathological analysis revealed papillary dermal oedema with neutrophilic infiltrate and scattered histiocytoid cells and eosinophils (Figure 2). There was minimal spongiosis, with no intraepidermal or subcorneal pustule formation and no evidence of vasculitis. A revised diagnosis of AGEP-like SS was made, attributed to aspiration pneumonia and recent antibiotic exposure. Histologically, it was consistent with the histiocytoid variant.

Clinical resolution was achieved 2 days after starting oral corticosteroids. An extensive malignancy screen was performed due to the strong association of histiocytoid SS with malignancy. This was negative except for positive faecal occult blood test and cancer antigen 19-9. A colonoscopy subsequently excluded an occult bowel malignancy and inflammatory bowel disease (IBD). He was reviewed 3 months later and was successfully weaned off prednisolone.

## Discussion

Pustular SS is a subtype of SS that typically presents as erythematous-based pustules.^1^ The AGEP-like presentation, as seen in our patient, probably represents a rare subset of pustular SS.

This case highlights the importance of recognizing SS as a clinical differential in patients with AGEP, given the differences in how the conditions are treated. AGEP is a self-limiting disease with management consisting of cessation of the causative agent combined with supportive care.^2^ Prednisolone is not typically used for management of AGEP as most patients recover after the causative medication is stopped.^2^ Prednisolone is typically required to treat SS.^3^

It is important to distinguish AGEP from SS given that SS has various associations with malignancy, infections, autoimmune diseases and medications.^3^ Histiocytoid SS is strongly associated with haematological malignancy, with up to 55% of patients having an associated haematological malignancy and 10% having a solid organ malignancy.^4^ In this case, our patient had an extensive malignancy screen to exclude an occult malignancy.

The cause of this patient’s presentation of AGEP-like SS was most likely a combination of aspiration pneumonia and recent antibiotic exposure, which included ceftriaxone and azithromycin, both of which are well-recognized triggers of SS.^5–7^ A drug rechallenge was not considered appropriate due to the severity of his presentation. An extended malignancy screen and rheumatological investigations were unremarkable, excluding recognized systemic associations. In addition, IBD was ruled out on colonoscopy.

We propose that AGEP-like SS is a rare manifestation of pustular SS. It is important to recognize SS as a differential for AGEP due to the differences in associations and treatment. Histiocytoid SS is a rare histological variant associated with haematological malignancies. Patients with this histological variant require vigilant investigations to rule out an underlying malignancy, and follow-up as cutaneous lesions can precede the diagnosis of haematological malignancy.^4^

## References

[1] Cohen PR . Sweet’s syndrome – a comprehensive review of an acute febrile neutrophilic dermatosis. Orphanet J Rare Dis 2007; 2:34. 17655751 10.1186/1750-1172-2-34 PMC1963326

[2] Feldmeyer L, Heidemeyer K, Yawalkar N. Acute generalized exanthematous pustulosis: pathogenesis, genetic background, clinical variants and therapy. Int J Mol Sci 2016; 17:1214. 27472323 10.3390/ijms17081214 PMC5000612

[3] Vashisht P, Goyal A, Hearth Holmes MP. Sweet syndrome. StatPearls. Available at: https://www.ncbi.nlm.nih.gov/books/NBK431050/ (last accessed 17 October 2025). 28613704

[4] Ghoufi L, Ortonne N, Ingen-Housz-Oro S et al Histiocytoid Sweet syndrome is more frequently associated with myelodysplastic syndromes than the classical neutrophilic variant. Medicine (Baltimore) 2016; 95:e3033. 27082547 10.1097/MD.0000000000003033 PMC4839791

[5] Rubegni P, Marano MR, Aloe GD et al Sweet’s syndrome and *Chlamydia pneumoniae* infection. J Am Acad Dermatol 2001; 44:862–4. 11312438 10.1067/mjd.2001.112580

[6] Nelson CA, Stephen S, Ashchyan HJ et al Neutrophilic dermatoses. J Am Acad Dermatol 2018; 79:987–1006. 29653210 10.1016/j.jaad.2017.11.064

[7] Costa JRC, Virgens AR, de Oliveira Mestre L et al Sweet syndrome: clinical features, histopathology, and associations of 83 cases. J Cutan Med Surg 2017; 21:211–16. 28300447 10.1177/1203475417690719

